# The Novel HDAC Inhibitor OBP-801 Promotes MHC Class I Presentation Through LMP2 Upregulation, Enhancing the PD-1-Targeting Therapy in Clear Cell Renal Cell Carcinoma

**DOI:** 10.3390/cancers16234058

**Published:** 2024-12-04

**Authors:** Tsukasa Narukawa, Shusuke Yasuda, Mano Horinaka, Keiko Taniguchi, Takahiro Tsujikawa, Mie Morita, Osamu Ukimura, Toshiyuki Sakai

**Affiliations:** 1 Department of Drug Discovery Medicine, Kyoto Prefectural University of Medicine, 465 Kajii-cho, Kawaramachi-Hirokoji, Kamigyo-ku, Kyoto 602-8566, Japan; tnarukaw@koto.kpu-m.ac.jp (T.N.); m-hori@koto.kpu-m.ac.jp (M.H.); ktaniguc@koto.kpu-m.ac.jp (K.T.); miemori@koto.kpu-m.ac.jp (M.M.); tsakai@koto.kpu-m.ac.jp (T.S.); 2 Department of Urology, Kyoto Prefectural University of Medicine, 465 Kajii-cho, Kawaramachi-Hirokoji, Kamigyo-ku, Kyoto 602-8566, Japan; ukimura@koto.kpu-m.ac.jp; 3 Department of Otolaryngology–Head & Neck Surgery, Kyoto Prefectural University of Medicine, 465 Kajii-cho, Kawaramachi-Hirokoji, Kamigyo-ku, Kyoto 602-8566, Japan; tu-ji@koto.kpu-m.ac.jp

**Keywords:** clear cell renal cell carcinoma, histone deacetylase inhibitor, immunoproteasome, major histocompatibility complex class I, immune checkpoint inhibitor, low molecular mass polypeptide 2, anti-PD-1 antibody, OBP-801

## Abstract

Background: Histone deacetylase (HDAC) inhibitors have been reported to exhibit immunomodulatory activities, including the upregulation of major histocompatibility complex class I (MHC class I). Although the immunoproteasome plays a pivotal role in MHC class I antigen presentation, its effect on immunotherapy for clear cell renal cell carcinoma (ccRCC) remains unclear. Methods: This study assessed whether OBP-801, a novel HDAC inhibitor, affects the expression of immunoproteasome subunits and subsequently the MHC class-I-mediated anti-cancer immunity in ccRCC. We analyzed the data of 531 patients with ccRCC from the Cancer Genome Atlas Kidney Clear Cell Carcinoma database. We further evaluated the treatment efficacy of the combination of OBP-801 and anti-PD-1 in a ccRCC mouse model. Results: Low molecular mass polypeptide (LMP) 2 was correlated most positively with CD3E, CD8A, and CD8B expression and estimated CD8^+^ T cell number. In vitro studies showed that OBP-801 upregulated MHC class I presentation by inducing LMP2 expression in the ccRCC cell lines RENCA, 786-O, and Caki-1. In vivo studies in a syngeneic mouse model with subcutaneous implantation of RENCA cells showed that OBP-801 treatment increased the percentage of CD45^+^CD3e^+^ T cells in tumor-infiltrating lymphocytes. The combination of anti-PD-1 antibody and OBP-801 enhanced the anti-tumor effect, LMP2 protein expression, and MHC class I presentation in tumor cells. MHC class I presentation in the tumors of each mouse was positively correlated with the percentage of CD45^+^CD3e^+^ T cells. Conclusions: Our results demonstrate that OBP-801 promotes MHC class I presentation through LMP2 upregulation in tumor cells and thereby potentiates PD-1-targeting therapy. These data suggest that the combination of OBP-801 and anti-PD-1 treatment is a promising therapeutic strategy for ccRCC.

## Simple Summary

Immune checkpoint inhibitors are used in combination with other drugs as the first-line therapy in the treatment of metastatic renal cell carcinoma. Although these cancer immunotherapies have demonstrated high therapeutic efficacy, half of patients develop treatment resistance. In this study, we investigated the enhancement of the antitumor effect of immunosuppressants by a histone deacetylase (HDAC) inhibitor. A novel HDAC inhibitor, OBP-801, upregulated LMP2, an immunoproteasome subunit, in clear cell renal cell carcinoma (ccRCC) cells, resulting in enhanced MHC class I expression, and a combined treatment of OBP-801 and anti-PD-1 antibody effectively alleviated ccRCC in mice. Our results suggest that manipulating LMP2 expression using OBP-801 may have therapeutic implications for enhancing MHC class I presentation and improving the anti-tumor immune response in ccRCC. In particular, the combination of OBP-801 with anti-PD-1 antibody appears to be promising for ccRCC treatment.

## 1. Introduction

Immune checkpoint inhibitors (ICIs), such as anti-PD-1/PD-L1 antibodies, are widely used in the treatment of various cancer types, including renal cell carcinoma (RCC) [1,2]. Although they have demonstrated high effectiveness, particularly in terms of achieving durable responses, their efficacy is limited to only a subset of patients [2,3]. Consequently, combination strategies with molecular targeted therapy have often been used to treat cancer patients resistant to immunotherapy [4,5,6]. In cancer immunity, antigen presentation to CD8^+^ T cells is mediated by major histocompatibility complex class I (MHC class I) molecules on the cell surface, and an elevated expression of MHC class I molecules generally promotes the activation of CD8^+^ T cells [7,8]. Tumor cells evade cancer immunity by inactivating this antigen processing machinery (APM) owing to the loss of MHC class I on the cell surface. This phenomenon has been observed in many cancer types, including RCC [9,10]. To unlock the maximum clinical potential of ICIs, it is crucial to prevent tumor escape, such as an immunosuppressive tumor microenvironment (TME).

Recently, histone deacetylase (HDAC) inhibitors have been reported to have anti-tumor effects, and some HDAC inhibitors, such as vorinostat, romidepsin, and panobinostat, have been approved by the United States Food and Drug Administration (FDA) for treating peripheral T cell lymphoma and multiple myeloma [11,12,13,14]. In addition to their effect of inducing cell cycle arrest and apoptosis, HDAC inhibitors are known to activate the APM in the TME and upregulate MHC class I presentation on the cell surface of tumors [14,15,16].

The immunoproteasome is a component of the APM that exchanges three catalytic subunits from the standard proteasome with low molecular weight polypeptide (LMP) 2, LMP7, and MECL-1, which alters the repertoire of peptides that are degraded. These peptides then bind to MHC class I molecules in the endoplasmic reticulum and are presented as antigens [7,17]. High immunoproteasome expression is associated with a better prognosis in patients with melanoma [18], and trichostatin A (TSA), an HDAC inhibitor, induces APM-related molecules and increases antigen presentation by MHC class I on the cell surface in melanoma [16]. Therefore, high expression of immunoproteasome may enhance CD8^+^ T-cell-mediated anti-tumor activity by upregulating antigen presentation via MHC class I.

OBP-801/YM753/spiruchostatin A is a bacteria-derived natural product containing a disulfide bond and has been identified as a p21-inducing agent using a p21 promoter reporter screen by us [19]. It was also shown that OBP-801 exerts potent class I HDAC inhibitory activity in tumor cells but not in normal cells [19]. Its anti-tumor effect against solid tumors in vivo has been previously reported [20,21,22], and its efficacy and safety for human use have been evaluated in a phase I trial [23]. However, the role of the immunoproteasome in RCC, and the mechanism of action of OBP-801 in tumor cells, remain unclear. Therefore, in the present study, we investigated the impact of OBP-801 on the expression of immunoproteasome subunits and its subsequent effect on MHC class-I-mediated anti-tumor immunity in clear cell RCC (ccRCC) cells. Furthermore, we evaluated the treatment effects of the combination of OBP-801 and anti-PD-1 antibody on a ccRCC mouse model.

## 2. Materials and Methods

### 2.1. Analysis of Clinically Available Transcriptomes and Tumor Immune Microenvironment

The normalized log2-transformed gene expression (RNA Seq V2 RSEM) of PSMB8, PSMB9, PSMB10, CD3E, CD8A, CD8B, and PTPRC (CD45) in 538 patients with ccRCC was obtained from The Cancer Genome Atlas Kidney Renal Clear Cell Carcinoma (TCGA-KIRC) using cBioPortal (https://www.cbioportal.org/, accessed on 5 March 2023). Next, the relative abundance of CD8^+^ T cells (described as the estimated CD8^+^ T cell number) in TCGA-KIRC patients was calculated using the xCell algorithm. After the integration of cBioPortal and xCell data, a dataset comprising 531 patients with ccRCC was used to investigate the correlation between the gene expression of immunoproteasome subunits and tumor immune microenvironment.

### 2.2. Cell Lines and Reagents

The mouse ccRCC cell line RENCA and human ccRCC cell lines 786-O and Caki-1 were obtained from the American Type Culture Collection (Rockville, MD, USA). RENCA and 786-O cells were maintained in RPMI-1640 (#05918; Nissui, Tokyo, Japan) with 10% (*v*/*v*) fetal bovine serum, 300 µg/mL glutamine, 50 units/mL penicillin G, and 100 µg/mL streptomycin. Caki-1 cells were maintained in EMEM (Wako, Osaka, Japan) supplemented with 10% (*v*/*v*) fetal bovine serum, 50 units/mL penicillin G, and 100 µg/mL streptomycin. Cells were incubated at 37 °C in a humidified atmosphere containing 5% CO_2_. OBP-801 was supplied by Oncolys BioPharma (Tokyo, Japan) and was dissolved in phosphate-buffered saline (PBS) at the indicated concentrations.

### 2.3. Western Blot Analysis

Cells were treated with PBS or the indicated concentrations of OBP-801 for 24 h and then lysed in lysis buffer (150 mmol/L NaCl, 50 mmol/L Tris-base, 1% NP-40, 0.5% sodium deoxycholate, and 0.1% SDS) supplemented with a phosphatase inhibitor cocktail mix (Nacalai Tesque, Kyoto, Japan) and a protease inhibitor cocktail mix (Nacalai Tesque). A 5× sample buffer (125 mmol/L Tris-base pH 6.8, 20% glycerol, 10% 2-mercaptoethanol, 4% SDS, and 0.04% bromophenol blue) was added to the lysate, and the mixtures were heated at 95 °C for 5 min. Equal amounts of the extracted proteins were separated by SDS-PAGE and transferred onto polyvinylidene difluoride membranes (Millipore, Billerica, MA, USA). Membranes were blocked using 5% fat-free dry milk in TBS with 0.1% Tween-20 and then incubated with primary and secondary antibodies in Signal Enhancer HIKARI (#02270-81; Nacalai Tesque). Proteins were visualized using Immobilon Western Chemiluminescent HRP Substrate (#WBKLS0500; Millipore), and chemiluminescence was detected using the ChemiDoc Touch imaging system (Bio-Rad Laboratories, Hercules, CA, USA). The primary antibodies included rabbit polyclonal PSMB9 antibody (1:1000, #14544-1-AP; Proteintech, Rosemont, IL, USA) and mouse monoclonal β-actin antibody (1:1000 or 1:2000, #A5441; Sigma-Aldrich, Saint Louis, MO, USA). HRP-linked donkey anti-rabbit IgG and HRP-linked sheep anti-mouse IgG were obtained from Cytiva (1:2000, #NA934 and #NA931, respectively; Tokyo, Japan). Densitometry was performed on Western blots using ImageJ 1.52a (National Institutes of Health, Bethesda, MD, USA). The protein levels were normalized for β-actin and presented as relative values to the level of control.

### 2.4. Reverse Transcription-Quantitative PCR (RT-qPCR) Analysis

RENCA cells were treated with PBS or the indicated concentrations of OBP-801 for 24, 48, or 72 h. For total RNA extraction, cells were lysed and purified using Sepasol-RNA I Super G (#09379-84; Nacalai Tesque) following the manufacturer’s protocol. cDNA was synthesized using the ReverTra Ace qPCR RT Master Mix (#FSQ-201; TOYOBO, Osaka, Japan). The synthesized cDNA was evaluated using THUNDERBIRD Probe qPCR Mix (#QPS-101; TOYOBO) and TaqMan Gene Expression Assays (Thermo Fisher Scientific, Waltham, MA, USA) using the QuantStudio 3 Real-Time PCR System (Thermo Fisher Scientific). PCR conditions were as follows: cycle 1, 50 °C for 2 min; cycle 2, 95 °C for 10 min; and cycle 3 (×40), 95 °C for 15 s and 60 °C for 1 min. Mouse-GAPDH (Mm99999915_g1; Thermo Fisher Scientific) and mouse-PSMB9 (Mm00479004_m1; Thermo Fisher Scientific) TaqMan probes were used for the experiments, and the gene expression level of *PSMB9* was normalized against that of GAPDH.

### 2.5. Analysis for the Cell Surface MHC Class I Expression

Cells were treated with PBS or the indicated concentrations of OBP-801 for 72 h. To prevent non-specific staining due to Fc receptor binding, RENCA cells were incubated on ice for 10 min with a CD16/CD32 monoclonal antibody (#14-0161-82; Thermo Fisher Scientific), and 786-O and Caki-1 cells were incubated on ice for 10 min with a Human Fc block (#564220; BD Biosciences, Franklin Lakes, NJ, USA). The samples were then divided into two groups: one group was treated with FITC-conjugated anti-MHC class I antibody (#11-5998-82; Thermo Fisher Scientific) or anti-HLA-ABC antibody (#11-9983-42; Thermo Fisher Scientific) and the other group was treated with FITC-conjugated control IgG (#11-4724-82; Thermo Fisher Scientific). All staining procedures were performed in a flow cytometry buffer (PBS supplemented with 2% BSA), and after incubation on ice for 20 min, the samples were analyzed using a BD Accuri C6 Plus flow cytometer (BD Biosciences). The data are presented as mean fluorescence intensity (MFI).

### 2.6. Transfection Experiments

Three stealth small interfering RNAs (siRNAs) targeting mouse *PSMB9* (#1: MSS275355, #2: MSS275356, and #3: MSS275357; Thermo Fisher Scientific) were used for RENCA cells. Silencer Select Pre-designed siRNAs targeting human *PSMB9* (#1: 4390824, siRNA ID: s11365, #2: 4390824, siRNA ID: s11366, and #3: 4392420, siRNA ID: s11367; Thermo Fisher Scientific) were used for 786-O and Caki-1 cells. Cells were incubated and transiently transfected with *PSMB9*-siRNA or negative-control-siRNA (#12935112; Thermo Fisher Scientific) using Lipofectamine RNAiMAX Transfection Reagent (#13778150; Thermo Fisher Scientific). After 24 h of transfection, the culture medium was replaced with fresh medium, and OBP-801 was added to each well. Western blot analysis was performed 24 h after OBP-801 addition to confirm the knockdown of *PSMB9*, and after 72 h of OBP-801 addition, flow cytometry was performed to confirm the effect of *PSMB9* knockdown on MHC class I expression.

### 2.7. Cell-Line-Derived Syngraft Mouse Study

The animal experiments in the present study were approved by the Committee for Animal Research of Kyoto Prefectural University of Medicine (permission no. M 2022-73) and performed according to the Institutional Animal Care and Use Committee guidelines. Five-week-old female BALB/c mice were purchased from Oriental Bioservice (Kyoto, Japan). After 1 week of acclimation, each mouse was injected subcutaneously in the right flank with RENCA cells (1 × 10^5^ cells/mouse) using high-concentration Matrigel (BD Biosciences). Nine days after implantation, 24 mice with an overall equal tumor volume were randomly divided into four groups (*n* = 5 in anti-PD-1 antibody group, *n* = 6 in other groups): vehicle, OBP-801, anti-PD-1 antibody, and combination of OBP-801 and anti-PD-1 antibody. Day 1 was considered as the day the treatment was started. OBP-801 (10 mg/kg) or 20% hydroxypropyl-β-cyclodextrin (HPCD, Nihon Syokuhin Kako Co., Tokyo, Japan) as a vehicle were injected into the tail vein of mice on days 1, 4, 7, and 11, and anti-PD-1 antibody (10 mg/kg, #BE0146; Bio X Cell, West Lebanon, NH, USA) or InVivoMAb polyclonal rat IgG (10 mg/kg, #BE0094; Bio X Cell) as non-reactive control IgG were injected intraperitoneally on days 1, 4, and 7. The tumor size of each mouse was measured three times a week, and the tumor volume was calculated from caliper measurements using the following formula: (length × width^2^)/2.

Mice were euthanized with intraperitoneal administration of sodium pentobarbital (200 mg/kg) on day 45 or when the tumor volume exceeded 1500 mm^3^. Survival analysis was used to compare the differences between groups according to survival time.

### 2.8. Western Blot and Flow Cytometry Analyses of Tumor Samples

RENCA cells (1 × 10^5^ cells/mouse) were implanted subcutaneously in the right flank of 5-week-old female BALB/c mice. After 11 days of implantation, 25 mice with approximately equal tumor volume were divided into four groups: vehicle (*n* = 5), OBP-801 (*n* = 5), anti-PD-1 antibody (*n* = 5), and combination (*n* = 10). The day treatment was started was defined as day 1, and the mice were injected with OBP-801 (10 mg/kg) or 20% HPCD on days 1, 4, and 7 and with anti-PD-1 antibody (10 mg/kg) or InVivoMAb polyclonal rat IgG (10 mg/kg) on days 1, 4, and 7. Tumor volume was measured every other day until day 8; mice were euthanized by intraperitoneal administration of sodium pentobarbital (200 mg/kg) on day 8. The death of mice was confirmed by lack of pulse, lack of breathing, and no response to toe pinch. The tumors were collected and dissociated into single-cell suspensions using a gentleMACSTM Octo Dissociator with Heaters (Miltenyi Biotec, Bergisch Gladbach, Germany). Western blotting and flow cytometry were performed for each sample. The procedure for Western blot analysis was the same as described above. For flow cytometry, cells were first blocked with a CD16/CD32 antibody (Thermo Fisher Scientific) and then stained with two different sets of antibodies to measure MHC class I expression and CD8^+^ T cells. Fixable Viability Stain 510 (FVS510; #564406; BD Biosciences), FITC-conjugated MHC class I antibody (#11-5998-82; Thermo Fisher Scientific), and APC-conjugated anti-CD45 antibody (17-0451-82; Fisher Scientific) were used for analyzing MHC class I molecules. FVS510, FITC-conjugated anti-mouse CD45 antibody (#553079; BD Biosciences), APC-conjugated anti-mouse CD3e antibody (#553066; BD Biosciences), PerCP-Cy5.5-conjugated anti-mouse CD8a antibody (#551162; BD Biosciences), and PE-Cyanine7-conjugated anti-mouse CD8b antibody (#126616; BioLegend, San Diego, CA, USA) were used for analyzing CD8^+^ T cells. Flow cytometry data were obtained using FACSCanto II (Becton-Dickinson, Franklin Lakes, NJ, USA), analyzed using the FlowJo version 10 software (BD Biosciences), and then presented as the rate of positive cell number or geometric mean of fluorescence intensity (gMFI). The gating strategy is shown in Figure S1. One researcher measured the samples by flow cytometry, and another performed the analysis of the data.

### 2.9. Cell Viability Assay

The number of viable cells for each ccRCC cell line (RENCA, 786-O, and Caki-1) was assessed using Cell Counting Kit-8 (CCK-8; Dojindo, Kumamoto, Japan). Cells were seeded and incubated with PBS or the indicated concentrations of OBP-801 for 72 h. The CCK-8 solution was added to each well, and the absorbance of the medium (450 nm) was determined using SpectraMax^®^ iD3 (Molecular Devices, LLC., San Jose, CA, USA) after a further 4 h of incubation. The percentage of growth was determined relative to that of cells treated with PBS. Experiments were repeated at least three times with triplicate samples.

### 2.10. Statistical Analysis

Each in vitro experiment was repeated at least two or three times, and data are shown as the mean ± standard deviation. The GraphPad Prism8 software (Dotmatics, San Diego, CA, USA) was used for statistical analysis, and the mean values of the two groups were compared using a two-tailed Student’s *t*-test. For comparing multiple groups, statistical significance was determined using one-way ANOVA. Pearson correlation statistics was employed for the analysis of correlation of a publicly available database and for in vivo correlation analysis. In in vivo experiments, the overall survival of each group was estimated by the Kaplan–Meier survival curve, and comparison among groups was made using stratified log-rank tests. The significance level was set at *p* < 0.05.

## 3. Results

### 3.1. Gene Expression of Immunoproteasome Subunits Is Associated with Tumor-Infiltrating CD8^+^ T Cells

**Figure 1:**
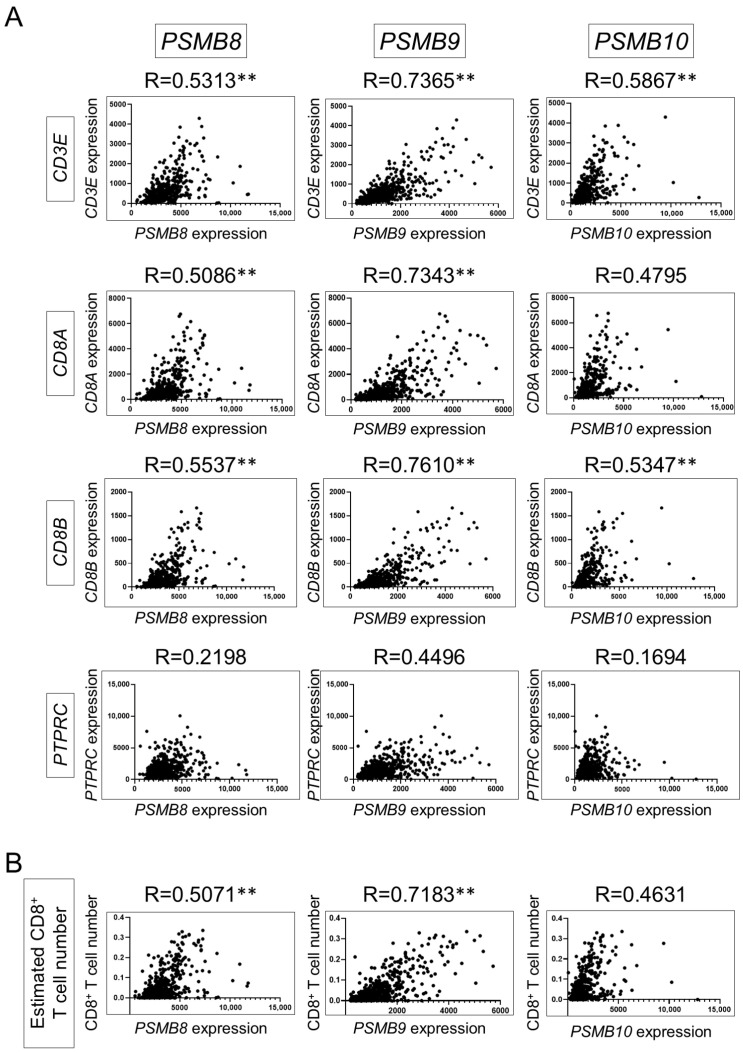
Among immunoproteasome subunits, the expression of Psmb9 in the TME was most strongly correlated with estimated CD8^+^ T cell number in ccRCC patients’ cohorts. Correlation of immunoproteasome subunits gene expression (PSMB8, PSMB9, and PSMB10) with CD3E, CD8A, CD8B, and PTPRC gene expression (**A**) and estimated CD8^+^ T cell number (**B**) in The Cancer Genome Atlas (TCGA) cohorts. Pearson correlation statistics were used for the analysis; ** R > 0.5.

MHC class I expression on the surface of tumor cells is associated with antigen presentation for CD8^+^ T cells. The immunoproteasome, one of the APM components, is involved in optimal antigen presentation of tumor epitopes to tumor-infiltrating CD8^+^ T cells [24,25]. Immunoproteasomes have a pro-tumorigenic or anti-tumorigenic role in the TME depending on the cancer type [18]. However, little is known about the role of proteasomes in ccRCC-specific anti-tumor immunity. To evaluate the association between the expression of each immunoproteasome subunit and CD8^+^ T-cell-mediated activity in ccRCC patients, the expression of PSMB8 (LMP7), PSMB9 (LMP2), PSMB10 (MECL-1), CD3E, CD8A, CD8B, and PTPRC (CD45) and estimated CD8^+^ T cell numbers were obtained from the TCGA database for 531 patients with ccRCC. We observed that immunoproteasome gene expression was significantly correlated with CD3E, CD8A, and CD8B gene expression and estimated CD8^+^ T cell numbers, except for PTPRC gene expression (Figure 1). Among all catalytic immunoproteasome subunits, LMP2 correlated most strongly with CD3E, CD8A, and CD8B gene expression and the estimated number of CD8^+^ T cells in this cohort.

### 3.2. Novel HDAC Inhibitor OBP-801 Upregulates LMP2 Expression and MHC Class I Presentation in ccRCC Cell Lines

**Figure 2:**
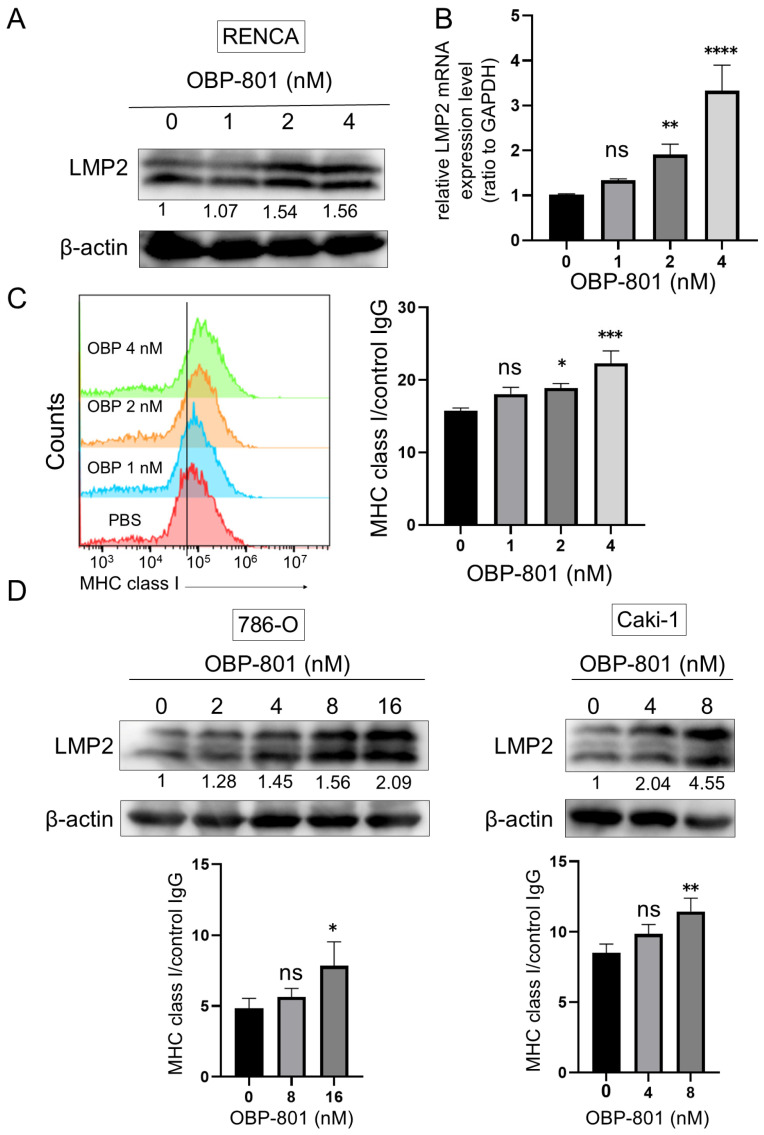
OBP-801 enhances expression of LMP2 (PSMB9) and increases MHC class I expression on the cell surface in ccRCC cell lines. (**A**,**D**) Western blotting was performed for LMP2 protein in RENCA, 786-O, and Caki-1 cells treated with OBP-801 at indicated concentrations for 24 h; β-actin was used as a loading control. The protein expression was quantified using ImageJ. The band intensity of LMP2 was corrected based on β-actin and presented as relative values to the level of control. (**B**) qRT-PCR was performed for LMP2 mRNA expression on RENCA cells treated with OBP-801 at indicated concentrations for 24 h in independent triplicate experiments; GAPDH was served as a loading control. (**C**,**D**) Expression of MHC class I molecules on the cell surface analyzed by flow cytometry in independent triplicate experiments. The cells were treated with OBP-801 at the indicated concentrations for 72 h. One-way ANOVA was used for statistical analysis of qRT-PCR and flow cytometry data; “ns” indicates not significant (*p* ≥ 0.05), * *p* < 0.05, ** *p* < 0.01, *** *p* < 0.001, **** *p* < 0.0001. Raw data and intensity measurements of Western blots in (**A**,**D**) are shown in Supplementary Figure S5.

We investigated the effect of OBP-801 on LMP2 expression in human and mouse ccRCC cell lines. First, the mouse ccRCC cell line, RENCA, was treated with OBP-801 for 24 h. LMP2 protein expression was upregulated by OBP-801 treatment (Figure 2A). Moreover, LMP2 gene expression was upregulated by OBP-801 in a dose-dependent manner at 24, 48, and 72 h (Figure 2B and Figure S2). To confirm the effect of OBP-801 on MHC class I expression on the surface of RENCA cells, flow cytometry was performed. As shown in Figure 2C, MHC class I expression on the cell surface was upregulated by OBP-801 in a dose-dependent manner for 72 h. The upregulation of LMP2 protein and cell surface MHC class I expression by OBP-801 was similarly observed in the human ccRCC cell lines 786-O and Caki-1 (Figure 2D). The concentration of OBP-801 in these experiments significantly suppressed the growth of these cell lines (Figure S3).

### 3.3. Knockdown of LMP2 Gene Downregulates MHC Class I Presentation in ccRCC Cell Lines

**Figure 3:**
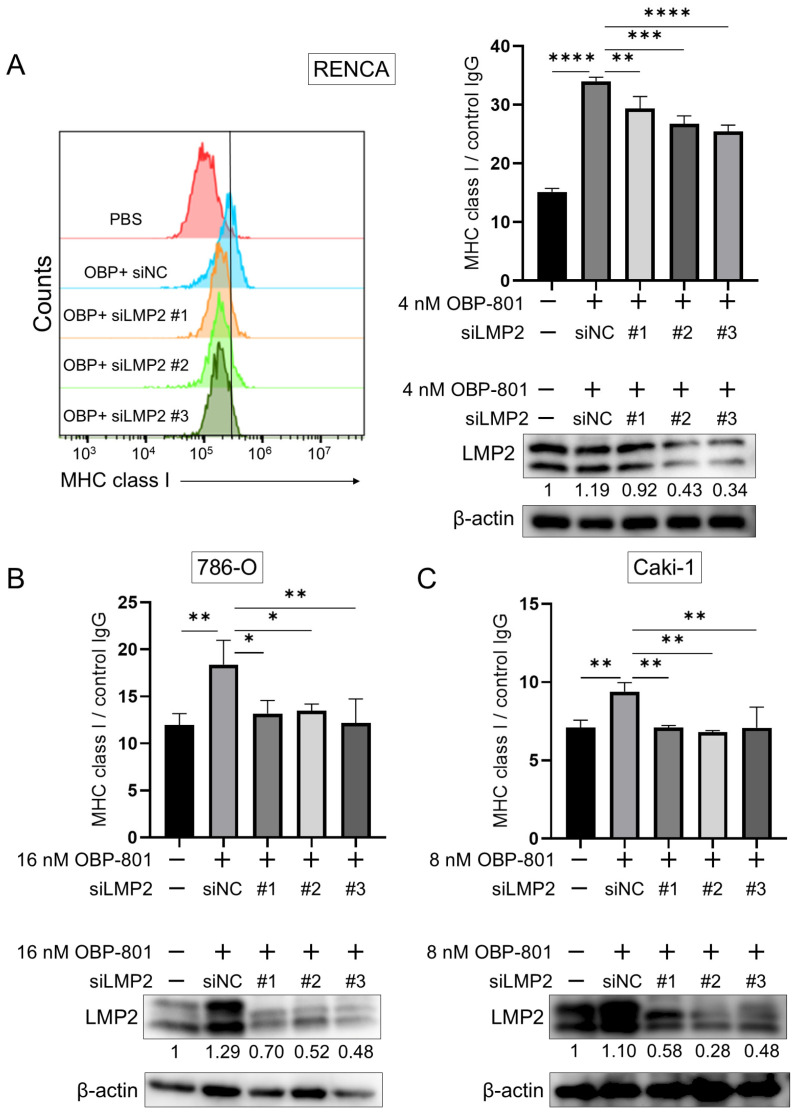
Knockdown of LMP2 by siRNA downregulates the elevated expression of MHC class I on the cell surface through OBP-801 administration. (**A**–**C**) RENCA, 786-O, and Caki-1 cells were incubated with siRNA for 24 h to knock down LMP2 and then treated with OBP-801 at the indicated concentrations. The treatment durations were 24 h for Western blotting and 72 h for flow cytometry. The LMP2 expression in these cells was evaluated by Western blotting; β-actin was served as a loading control. The protein expression was quantified using ImageJ. The band intensity of LMP2 was corrected based on β-actin and presented as relative values to the level of control. The expression of MHC class I molecules on the cell surface was analyzed by flow cytometry in independent triplicate experiments. One-way ANOVA was used for the statistical analysis of flow cytometry data; * *p* < 0.05, ** *p* < 0.01, *** *p* < 0.001, **** *p* < 0.0001. Raw data and intensity measurements of Western blots are shown in Supplementary Figure S6.

To determine whether the enhancement of LMP2 expression by OBP-801 affected MHC class I expression on the cell surface, we performed gene knockdown of LMP2 using siRNA for each cell line. The suppression of LMP2 protein expression by si-LMP2 was confirmed by Western blot analysis, and the effect of LMP2 knockdown on MHC class I presentation was confirmed by flow cytometry. In all three cell lines, reduction in LMP2 protein downregulated MHC class I expression on the cell surface (Figure 3 and Figure S5). This suggests that LMP2, one of the APM components, can regulate MHC class I expression on the cell surface, at least in the presence of OBP-801.

### 3.4. OBP-801 Enhances the Anti-Tumor Activity of PD-1 Inhibition in RENCA Allograft Model

**Figure 4:**
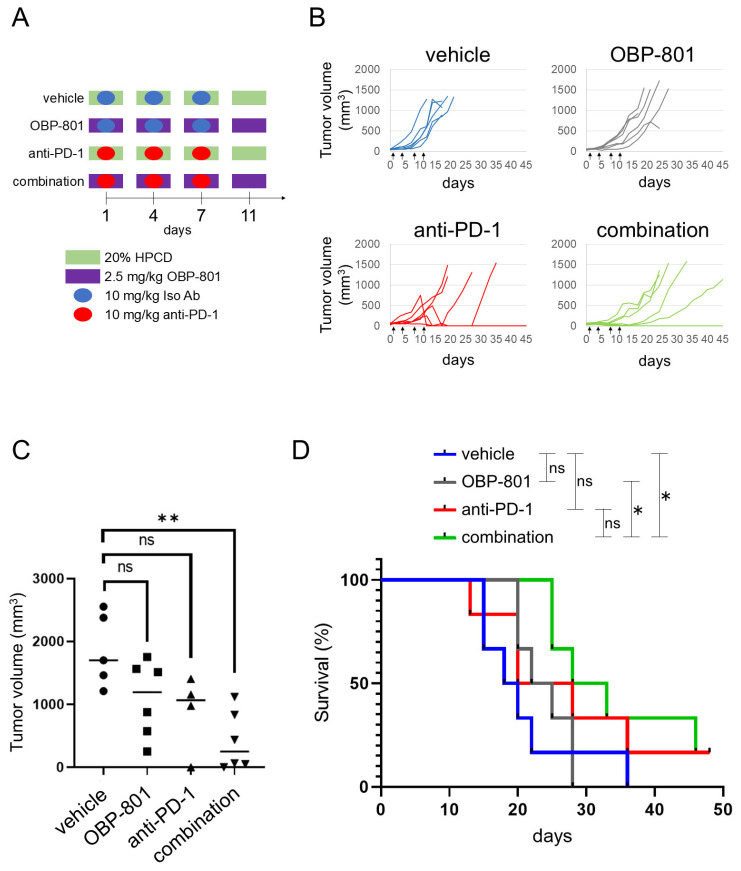
Combination of OBP-801 and anti-PD-1 antibody treatment leads to increasing anti-tumor activity compared with single-agent treatment. (**A**) Treatment timeline of mice implanted with RENCA cells. (**B**) Individual tumor volumes in each treatment group over time (*n* = 6 per group). (**C**) Individual tumor volumes on day 15. One-way ANOVA was used for statistical analysis; “ns” indicates not significant (*p* ≥ 0.05), ** *p* < 0.01. (**D**) Survival plot for each treatment group. The log-rank test was used for the statistical analysis of each survival curve; “ns” indicates not significant (*p* ≥ 0.05), * *p* < 0.05.

The upregulation of MHC class I expression on the cell surface of tumors may enhance CD8^+^ T-cell-mediated anti-tumor activity following anti-PD-1 treatment. To assess the therapeutic effect of the combination of anti-PD-1 antibody and OBP-801 in vivo, we performed anti-tumor studies in a RENCA tumor-bearing BALB/c mouse model. We selected a small-sized experiment to examine the effects of combination therapy as an initial experiment. We randomly divided 24 RENCA-allografted mice into four groups based on tumor volume (mm^3^) (*n* = 5 in anti-PD-1 antibody group, *n* = 6 in other groups): vehicle, OBP-801, anti-PD-1 antibody, and a combination of OBP-801 and anti-PD-1 antibody. The treatment timeline is shown in Figure 4A. Sequential changes in the tumor volume in each mouse according to the treatment groups are shown in Figure 4B. The tumor volumes after 15 days of treatment were compared among the treatment groups (Figure 4C). The combination of OBP-801 and anti-PD-1 antibody treatment was most effective in enhancing tumor growth inhibition (*p* = 0.0029, 0.18, and 0.10 for vehicle vs. combination, OBP-801, and anti-PD-1, respectively).

To evaluate the effect of the combination treatment on mouse survival, survival plots for each group were generated (Figure 4D). A significant increase in mouse survival was observed in the combination treatment group compared with the OBP-801 and vehicle groups (*p* = 0.026 and 0.03, respectively). However, comparisons among the other group demonstrated that the vehicle and OBP-801 or anti-PD-1 alone provided no significant survival benefits. Our results showed that OBP-801 and anti-PD-1 mutually enhanced each other’s effects.

### 3.5. Combination of OBP-801 and Anti-PD-1 Antibody Treatment Upregulates MHC Class I Expression on Tumor Cells and Is Correlated with Intratumoral T Cell Abundance with a Reduction in Tumor Growth

**Figure 5:**
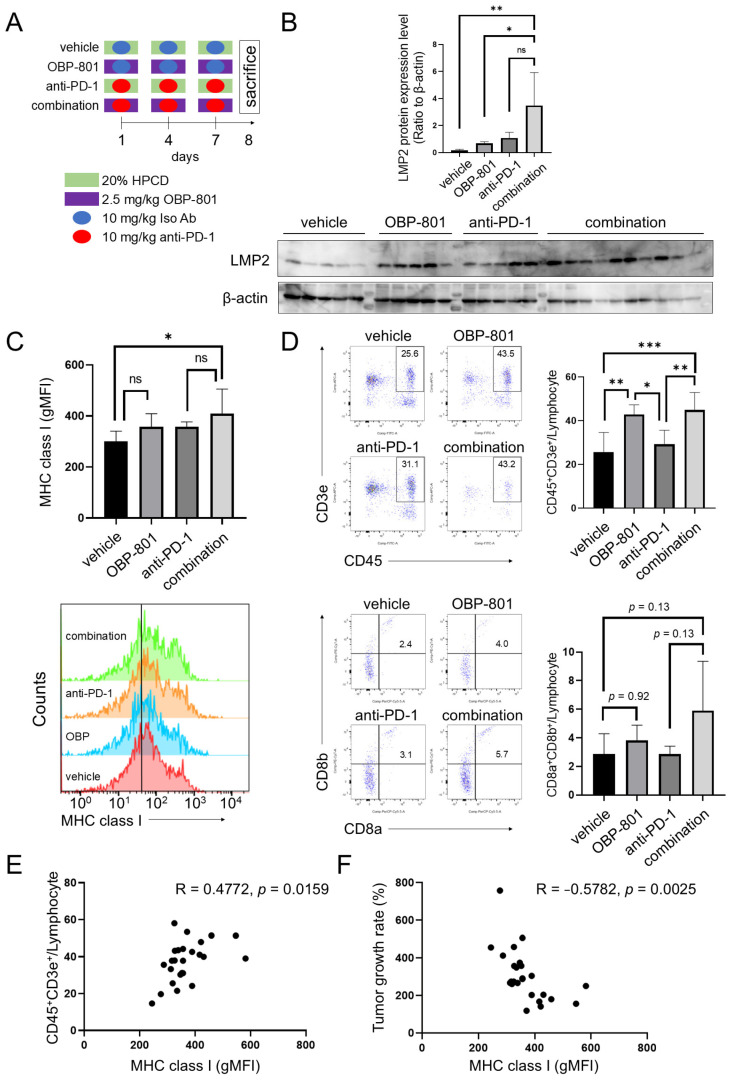
Combination of OBP-801 and anti-PD-1 antibody treatment upregulates MHC class I expression on the cell surface, resulting in elevated T cells in TILs and a reduction in tumor growth rate. (**A**) Treatment timeline for mice implanted with RENCA. (**B**) Western blotting was performed to determine the individual expression of the LMP2 protein in each group (vehicle, *n* = 5; OBP-801, *n* = 5; anti-PD-1 antibody, *n* = 5; and combination, *n* = 10). β-actin was served as a loading control. Protein expression was quantified and determined using ImageJ, and band intensities of LMP2 were corrected in reference to β-actin. One-way ANOVA was used for statistical analysis; “ns” indicates not significant (*p* > 0.05), * *p* < 0.05, ** *p* < 0.01. (**C**) Individual MHC class I expression on the cell surface of each group was analyzed using flow cytometry. One-way ANOVA was used for statistical analysis; “ns” indicates not significant (*p* > 0.05), * *p* < 0.05. (**D**) Percentages of T cells (CD45^+^ CD3e^+^) and CD8^+^ T cells in TIL from each group were analyzed using flow cytometry. The dot plots of each median sample in all groups are shown. One-way ANOVA was employed for the statistical analysis of the percentage of T cells and percentage of CD8^+^ T cells; * *p* < 0.05, ** *p* < 0.01, *** *p* < 0.001. (**E**,**F**) Correlation of individual MHC class I expression with percentage of T cells and tumor growth rate (%, tumor volume on day 15/tumor volume on day 1) in each mouse. Pearson correlation coefficients were used for statistical analysis. Raw data and intensity measurements of Western blots are shown in Supplementary Figure S7.

We used the RENCA allograft mouse model to clarify whether the inhibition of tumor growth by the combination of OBP-801 and anti-PD-1 antibody treatment was associated with enhanced antigen presentation and/or immune response. The treatment schedule is shown in Figure 5A. Tumor size was measured on day 8 after the treatment began, and after harvesting the tumors, each sample was examined for the expression of LMP2 protein, MHC class I molecules on the cell surface, and tumor-infiltrating lymphocytes (TILs) (Figure S4). LMP2 expression was significantly higher in the combination group than in the vehicle or OBP-801 group (*p* = 0.006 and *p* = 0.021, respectively; Figure 5B). A similar trend was observed for MHC class I expression on the cell surface—it was significantly higher for the combination group than for the vehicle group (*p* = 0.024; Figure 5C).

Expression of lymphocyte T cell (CD45^+^, CD3e^+^) populations was confirmed by flow cytometry. In the TME, CD8^+^ (CD8a^+^CD8b^+^) T cells are known to be differentiated from lymphocyte T cells (CD45^+^, CD3e^+^), and that has a key role in anti-tumor immunity. In the TILs collected in this study, the proportion of CD45^+^CD3e^+^ cells was significantly increased by OBP-801 with or without anti-PD-1 antibody treatment (*p* = 0.007: vehicle vs. OBP-801, *p* = 0.04: OBP-801 vs. anti-PD-1 antibody, *p* = 0.005: anti-PD-1 antibody vs. combination, *p* = 0.0006: vehicle vs. combination). The proportion of CD8a^+^CD8b^+^ cells was also increased by OBP-801 with anti-PD-1 antibody, but the difference was not statistically significant (Figure 5D). Next, to detect the correlation between MHC class I expression on the cell surface and the proportion of CD45^+^CD3e^+^ cells or tumor growth rate (tumor volume on day 8/tumor volume on day 1), we performed Pearson correlation statistics. MHC class I presentation was positively correlated with the proportion of CD45^+^CD3e^+^ cells and negatively correlated with tumor growth rate (*p* = 0.0159 and *p* = 0.0025, respectively; Figure 5E,F). These results suggest that OBP-801 enhances MHC class-I-mediated antigen presentation as well as anti-PD-1 immunotherapy.

## 4. Discussion

According to GLOBOCAN 2018 data, RCC is the seventh most common neoplasm worldwide, accounting for approximately 2% of all cancer diagnoses and deaths. The incidence of this disease has increased in developed countries over the past decades and has more than doubled since 1975 in the United States [26,27].

ICIs, including anti-PD-1/PD-L1 antibodies, are used in combination with other agents as first-line therapy for treating metastatic RCC and may also be used as adjuvant therapy after surgery [6,28,29]. Although these cancer immunotherapies have demonstrated high therapeutic efficacy, including durable responses and certain complete response rates, half of the patients develop resistance to treatment after 11.6–23.9 months [30,31,32,33,34].

Downregulation of antigen presentation due to loss of MHC class I molecules is one of the acquired immune resistance mechanisms after ICI administration [3,35], and APM is known to be involved in MHC class I presentation. Analysis of the TCGA database also showed that the three catalytic subunits of the immunoproteasome were associated with CD3E, CD8A, and CD8B expressions and estimated CD8^+^ T cell number. In contrast, HDAC inhibitors can potentially reverse cell resistance to tumor immunotherapy by upregulating MHC class I molecules on the cell surface [4,8,15,16,36]. HDAC inhibitors, with or without pre-administration of ICIs, can enhance MHC class I expression in the TME. In combination with anti-PD-1/PD-L1 treatment, they can increase tumor-infiltrating CD8^+^ T cells and the CD8^+^/regulatory T cell ratio and decrease myeloid-derived suppressor cells (MDSCs) in the TME [37,38,39,40,41,42,43,44].

Similar to previous reports on other HDAC inhibitors, the present study also found that MHC class I presentation was upregulated by OBP-801 in a dose-dependent manner, and this upregulation was suppressed by LMP2 knockdown in all ccRCC cell lines. The results indicated that MHC class I presentation is partially regulated by LMP2 and that OBP-801 enhances MHC class I presentation by upregulating LMP2.

Immunoproteasomes have positive or negative effects on tumorigenesis depending on the cancer type [18,45]. In this study, the TCGA database results showed that LMP2 expression was associated with the population of CD8^+^ T cells in tumors, and in vivo studies suggested that MHC class I presentation with LMP2 expression enhanced anti-tumor activity. Hence, LMP2 upregulation possibly promotes the efficacy of cancer immunotherapy in patients with ccRCC.

Furthermore, in the RENCA allograft mouse model, combination treatment significantly increased LMP2 expression, MHC class I expression on the cell surface, and the percentage of CD45^+^ CD3e^+^ T cells (Figure 5) and reduced tumor volume (Figure 4). Although the proportion of CD8a^+^CD8b^+^ cells tended to increase under the combination treatment, the difference was not significant. This may be due to differences in the effectiveness of anti-PD-1 antibody among individual mice. As shown in Figure 4B, the tumor growth in mice tended to be more varied in the anti-PD-1 antibody treatment group and the combination group compared to the vehicle and OBP-801 single agent groups, suggesting that immunological responses likely differ among mice. Hence, it may be necessary to pay attention to these individual differences when studying the anti-tumor effect of immunity, even in mice with the same genetic background. Although it is unavoidable to maintain the 3R principle, the small number of mice in each group is a limitation of this study. MHC class I presentation was positively correlated with the proportion of CD45^+^CD3e^+^ cells and negatively correlated with the tumor growth rate (Figure 5E,F).

**Figure 6:**
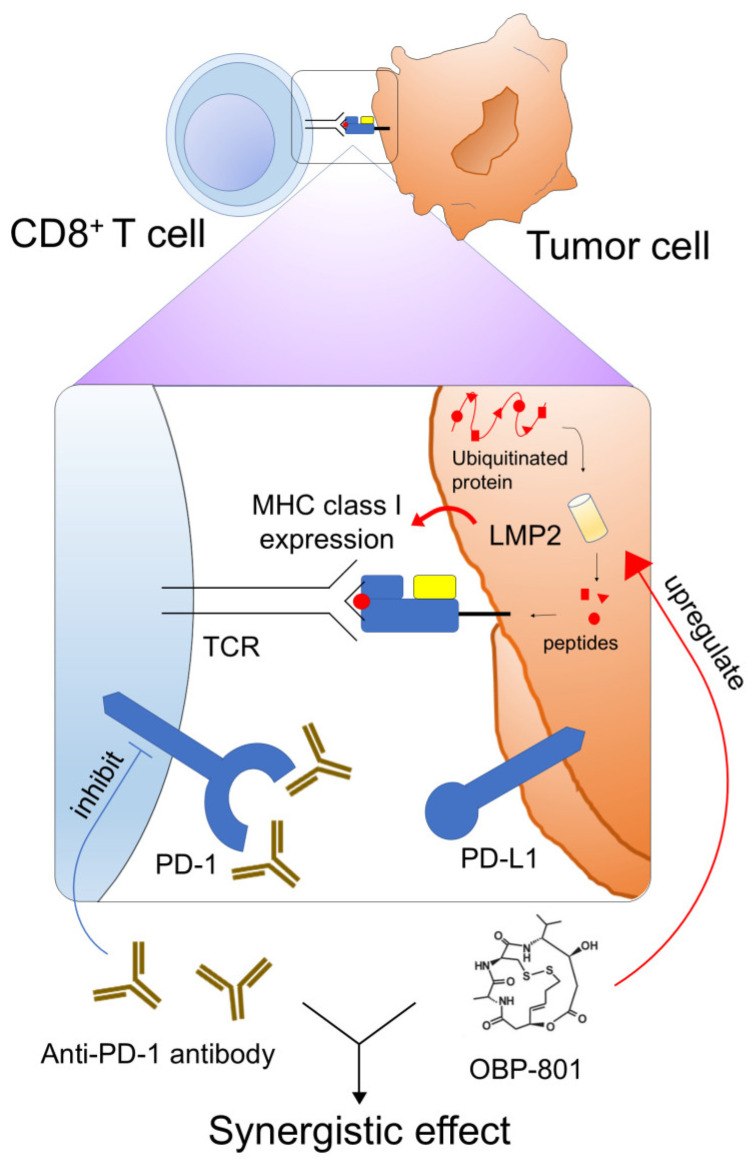
Schematic overview showing the synergistic effect between OBP-801 and anti-PD-1 antibody treatment. OBP-801 upregulates LMP2 expression and increases MHC class I antigen expression. Upregulation of MHC class I on the tumor cell surface increases the number of T cells (CD45^+^CD3e^+^) in TILs. In contrast, PD-1 (PD-L1) expression is one of the major mechanisms of cancer immune evasion, and anti-PD-1 (PD-L1) antibody treatment increases the anti-tumor activity of CD8^+^ T cells. Hence, the combination of OBP-801 and an anti-PD-1 antibody treatment creates a synergistic effect in ccRCC, leading to optimal anti-tumor immunity.

In summary, our results suggest that OBP-801 upregulates LMP2, resulting in enhanced MHC class I expression on the surface of ccRCC cells, and combined treatment with OBP-801 and anti-PD-1 antibody creates a synergistic effect with possibly enhanced immunity (Figure 6). To the best of our knowledge, this is the first study to show the effect of LMP2 on MHC class I presentation and the treatment efficacy of an LMP2 inducer in combination with anti-PD-1 antibody.

ccRCC is the most common histological subtype of RCC, and its occurrence is associated with mutations in the von Hippel–Lindau (VHL) gene. The mutated VHL gene accumulates intracellular hypoxia-inducible factor (HIF), resulting in the activation of hypoxia-inducible genes, including vascular endothelial growth factor (VEGF) [46].

Before the approval of ICIs, the major systemic therapy for metastatic RCC was anti-angiogenic drugs targeting VEGF or its receptors [46]. HIF activity is inhibited by class I HDAC inhibitors [47], and cell invasion in ccRCC is increased by HDAC 1 [48]. OBP-801, a potent class I HDAC inhibitor, is also expected to be effective in inhibiting HIF activity and ccRCC cell invasion. OBP-801 exerts its anti-tumor effects on tumor cells [19] primarily by inducing apoptosis and cell cycle arrest in various solid tumors [20,21,22], indicating that OBP-801 has potential application in anti-cancer therapy.

Recently, HDAC inhibitors have been reported to upregulate MHC class II15 and MHC class I molecules. PD-L1 acetylation is associated with the evasion of immune surveillance in tumor cells, and HDAC inhibitors may enhance tumor immunotherapy by targeting PD-L1 acetylation [49].

## 5. Conclusions

Among various mechanisms involved in cancer immunity, the PD-1/PD-L1 interaction is a major immune escape mechanism [50]. OBP-801 may promote MHC class I presentation by inducing LMP2 expression and enhancing anti-tumor immunity via anti-PD-1/PD-L1 antibody treatment. Our results suggest that the combination of OBP-801 and anti-PD-1/PD-L1 antibody treatment is a promising therapy for ccRCC.

## Data Availability

Data are contained within the article or Supplementary Materials.

## Supplementary Materials

The following supporting information can be downloaded at: https://www.mdpi.com/article/10.3390/cancers16234058/s1, Figure S1: Flow cytometric analysis; Figure S2: RT-qPCR for RENCA in 48 and 72 h; Figure S3: Cell viability assay for each cell line; Figure S4: The pictures of tumor tissue; Figure S5: Uncropped Western blot images presented in Figure 2; Figure S6: Uncropped Western blot images presented in Figure 3; Figure S7: Uncropped Western blot images presented in Figure 5.
