# Supplementary material for: The Novel HDAC Inhibitor OBP-801 Promotes MHC Class I Presentation Through LMP2 Upregulation, Enhancing the PD-1-Targeting Therapy in Clear Cell Renal Cell Carcinoma
Source: Cancers (Basel). 2024 Dec 4;16(23):4058. doi: 10.3390/cancers16234058 (PMC11640654; doi:10.3390/cancers16234058)
Supplement: Supplementary file 1 [file cancers-16-04058-s001.zip › cancers-3325730-supplementary.pdf]

Supplementary files

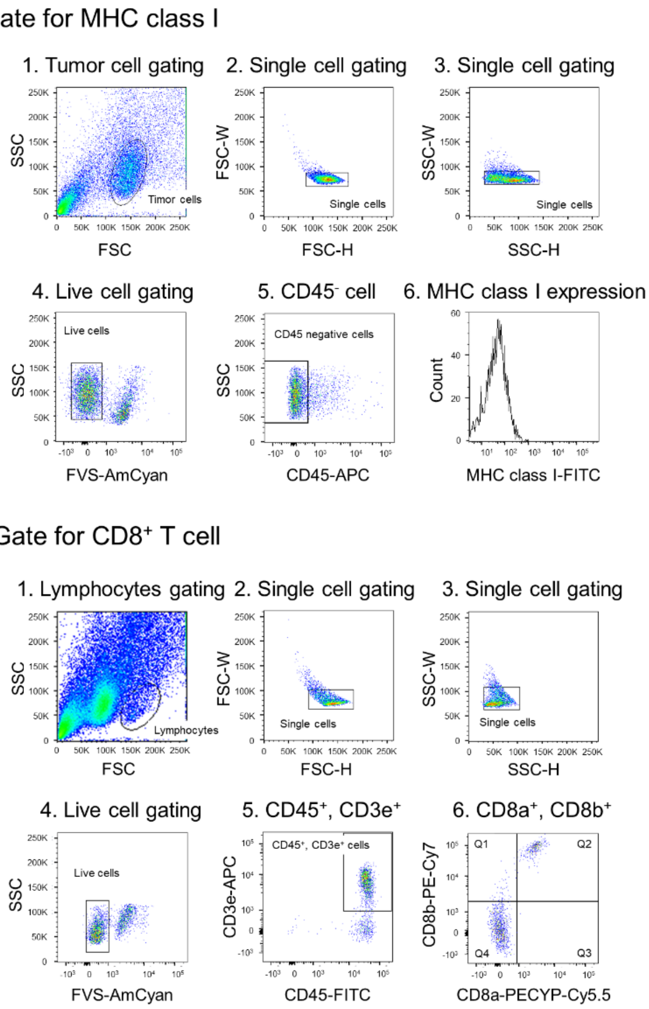

Figure S1. Flow cytometric analysis.

Gate for MHC class I; RENCA cells were gated using forward scatter (FSC) vs side scatter (SSC) dot plot. After removal of doublets using FSC-H vs FSC-W plot and SSC-H vs SSC-W plot, the AmCyan$^-$ cells were defined as viable cells. CD45 non-expressing cells were defined as tumor cells and MHC class I expression was measured.

Gate for CD8$^+$ T cells; Lymphocytes were gated using FSC vs SSC dot plot. After

removal of doublets using FSC-H vs FSC-W plot and SSC-H vs SSC-W plot, the AmCyan^{-} cells were defined as viable cells. CD45^{+}, CD3e^{+} cells were defined as T cells, and cells expressing CD8a and CD8b were measured as CD8^{+} T cells.

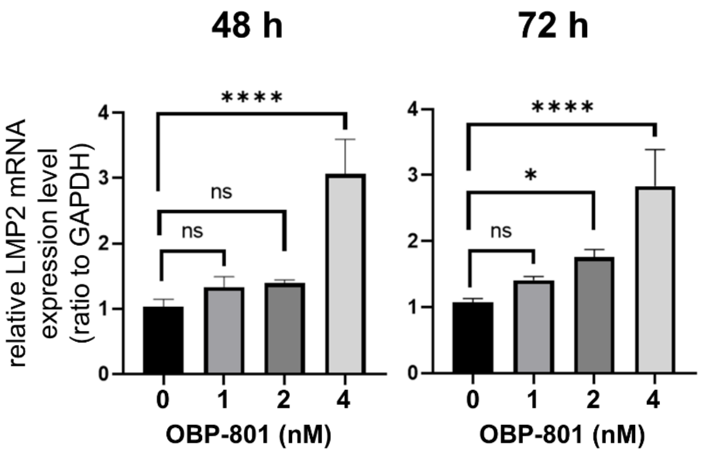

Figure S2. RT-qPCR for RENCA in 48 and 72 hours

qRT-PCR was carried out for LMP2 mRNA expression on RENCA cells treated with OBP-801 as indicated concentrations for 48 and 72 hours in independent triplicates experiments; The expression of each mRNA was normalized to that of GAPDH mRNA in the same sample. * $p < 0.05$, **** $p < 0.0001$.

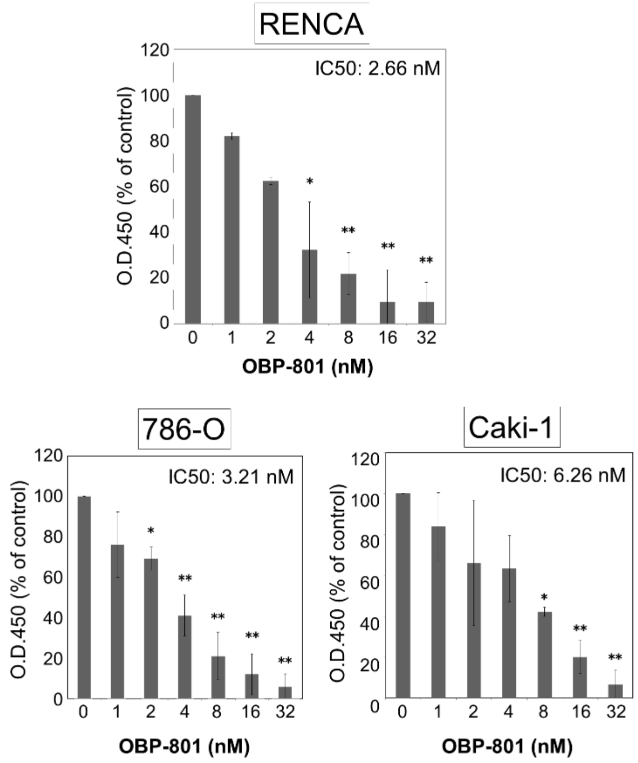

Figure S3. Cell viability assay for each cell line

ccRCC cell lines (RENCA, 786-O, Caki-1) were assessed with Cell Counting Kit-8 after treatment with OBP-801 as indicated concentrations for 72 hours. The IC_{50} values were determined for each cell line. The bars indicate the mean ± standard deviation (SD) values for independent experiments performed in triplicate. * $p < 0.05$, ** $p < 0.01$.

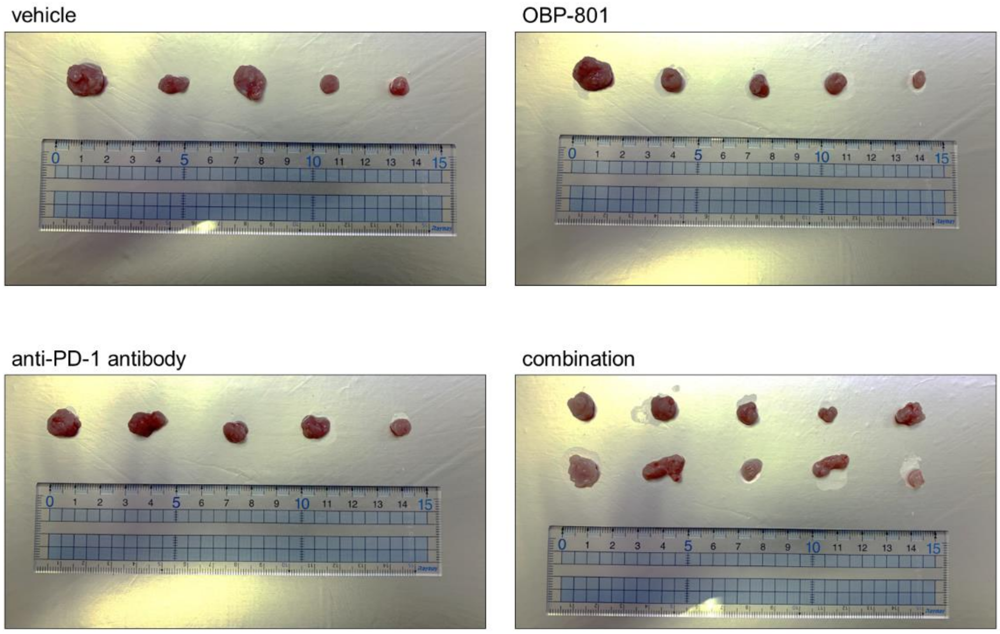

Figure S4. The pictures of tumor tissue

The photos of tumor tissues removed 8 days after the start of treatment in the syngraft mouse study shown in Figure 5.

A. Renca

lane 1: 0 nM OBP-801
lane 2: 1 nM OBP-801
lane 3: 2 nM OBP-801
lane 4: 4 nM OBP-801

lane	1	2	3	4
LMP2	1.00	0.85	1.11	1.15
β-actin	1.00	0.80	0.72	0.74
LMP2/β-actin	1.00	1.07	1.54	1.56

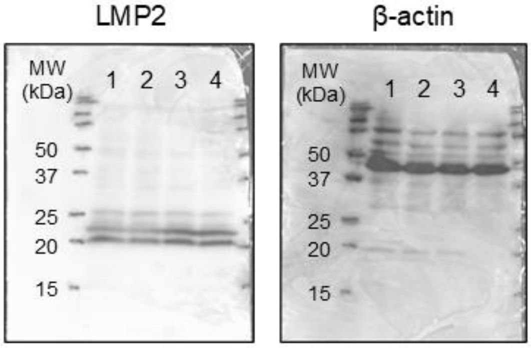

B. 786-O

lane 1: 0 nM OBP-801
lane 2: 2 nM OBP-801
lane 3: 4 nM OBP-801
lane 4: 8 nM OBP-801
lane 5: 16 nM OBP-801

lane	1	2	3	4	5
LMP2	1.00	1.36	1.71	1.86	2.21
β-actin	1.00	1.06	1.18	1.19	1.06
LMP2/β-actin	1.00	1.28	1.45	1.56	2.09

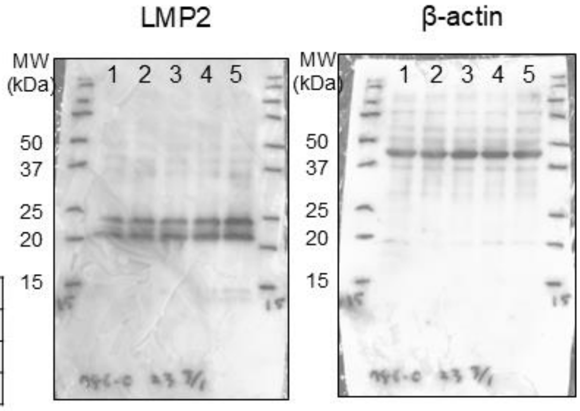

C. Caki-1

lane 1: 0 nM OBP-801
lane 2: 4 nM OBP-801
lane 3: 8 nM OBP-801

lane	1	2	3
LMP2	1.00	1.72	2.24
β-actin	1.00	0.85	0.49
LMP2/β-actin	1.00	2.04	4.55

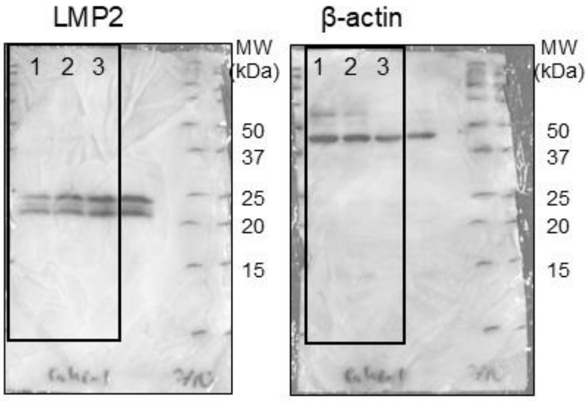

Figure S5. Uncropped Western blot images presented in Figure 2

Molecular weight (MW) markers are shown on the side of the images. The band intensities of LMP2 protein and β-actin protein relative to lane 1 (0 nM OBP-801) and the expression ratio of LMP2 corrected by β-actin are shown in the table.

A. Renca

lane 1: control
lane 2: 4 nM OBP-801 + siNC
lane 3: 4 nM OBP-801 + siLMP2 #1
lane 4: 4 nM OBP-801 + siLMP2 #2
lane 5: 4 nM OBP-801 + siLMP2 #3

lane	1	2	3	4	5
LMP2	1.00	0.95	0.87	0.42	0.38
β-actin	1.00	0.80	0.94	0.96	1.13
LMP2/β-actin	1.00	1.19	0.92	0.43	0.34

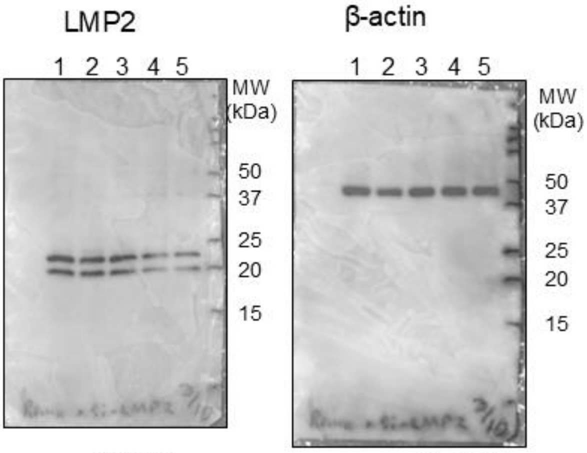

B. 786-O

lane 1: control
lane 2: 16 nM OBP-801 + siNC
lane 3: 16 nM OBP-801 + siLMP2 #1
lane 4: 16 nM OBP-801 + siLMP2 #2
lane 5: 16 nM OBP-801 + siLMP2 #3

lane	1	2	3	4	5
LMP2	1.00	1.71	0.60	0.55	0.33
β-actin	1.00	1.33	0.86	1.07	0.70
LMP2/β-actin	1.00	1.29	0.70	0.52	0.48

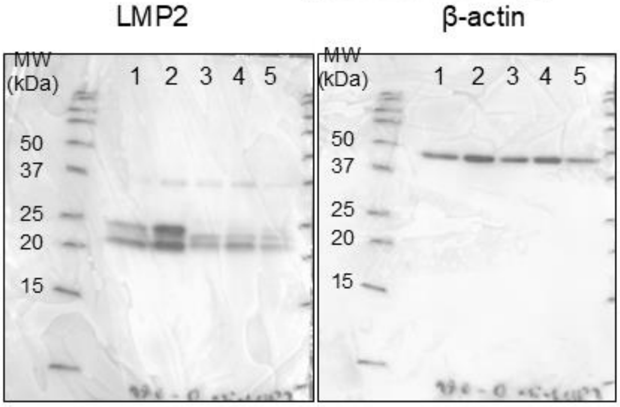

C. Caki-1

lane 1: control
lane 2: 8 nM OBP-801 + siNC
lane 3: 8 nM OBP-801 + siLMP2 #1
lane 4: 8 nM OBP-801 + siLMP2 #2
lane 5: 8 nM OBP-801 + siLMP2 #3

lane	1	2	3	4	5
LMP2	1.00	1.27	0.70	0.40	0.59
β-actin	1.00	1.15	1.20	1.42	1.23
LMP2/β-actin	1.00	1.10	0.58	0.28	0.48

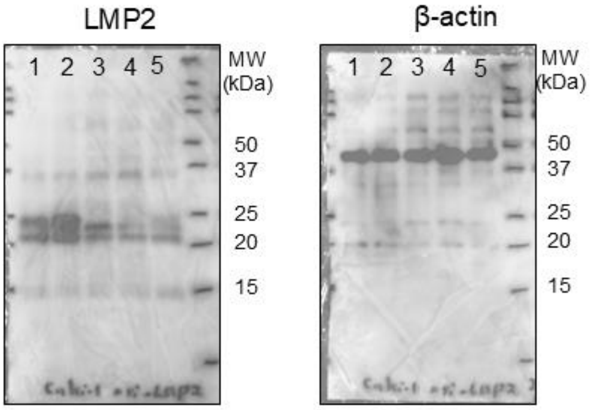

Figure S6. Uncropped Western blot images presented in Figure 3

Molecular weight (MW) markers are shown on the side of the images. The band intensities of LMP2 protein and β-actin protein relative to lane 1 (control) and the expression ratio of LMP2 corrected by β-actin are shown in the table.

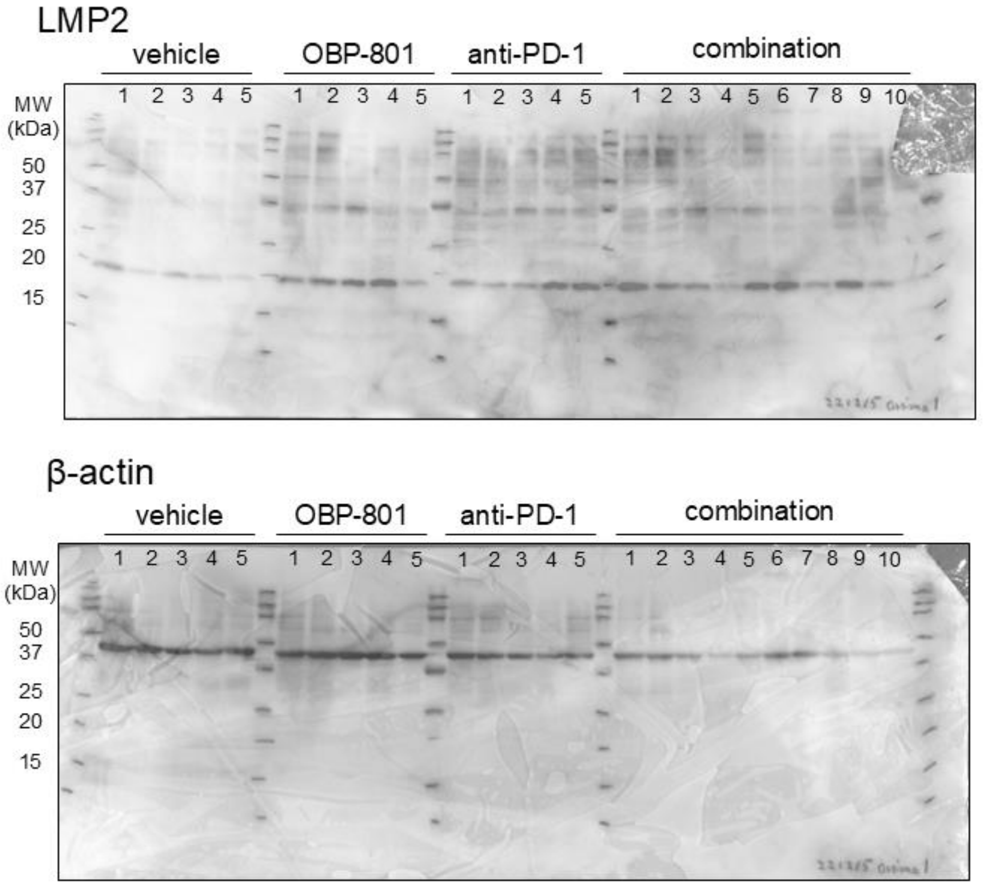

	Vehicle					OBP-801					anti-PD-1				
lane	1	2	3	4	5	1	2	3	4	5	1	2	3	4	5
LMP2	1.00	0.11	0.41	0.51	0.86	2.51	2.93	2.66	3.68	1.97	2.36	1.87	2.58	3.73	3.92
β-actin	1.00	0.76	0.52	0.56	0.75	0.85	0.87	0.78	0.82	0.70	0.75	0.56	0.48	0.49	0.52
LMP2/β-actin	1.00	0.14	0.79	0.91	1.15	2.93	3.37	3.39	4.46	2.82	3.14	3.31	5.39	7.54	7.56

	combination									
lane	1	2	3	4	5	6	7	8	9	10
LMP2	5.01	3.41	3.17	3.13	4.01	4.57	2.99	3.79	3.12	3.24
β-actin	0.47	0.39	0.20	0.06	0.19	0.36	0.39	0.33	0.18	0.19
LMP2/β-actin	10.63	8.74	15.82	49.25	21.55	12.57	7.63	11.64	17.54	17.46

Figure S7. Uncropped Western blot images presented in Figure 5

Molecular weight (MW) markers are shown on the left side of the images. The band intensities of LMP2 protein and β-actin protein relative to lane 1 (Vehicle) and the expression ratio of LMP2 corrected by β-actin are shown in the table.
